# Hypoxic environment of wounds and photosynthesis-based oxygen therapy

**DOI:** 10.1093/burnst/tkae012

**Published:** 2024-06-10

**Authors:** Shuning Tian, Shenyu Tan, Mingjie Fan, Wenlin Gong, Tianchang Yang, Fangwen Jiao, Hongzhi Qiao

**Affiliations:** Jiangsu Engineering Research Center for Efficient Delivery System of TCM, School of Pharmacy, Nanjing University of Chinese Medicine, 138 Xianlin Avenue, Qixia District, Nanjing 210023, China; Jiangsu Engineering Research Center for Efficient Delivery System of TCM, School of Pharmacy, Nanjing University of Chinese Medicine, 138 Xianlin Avenue, Qixia District, Nanjing 210023, China; Jiangsu Engineering Research Center for Efficient Delivery System of TCM, School of Pharmacy, Nanjing University of Chinese Medicine, 138 Xianlin Avenue, Qixia District, Nanjing 210023, China; Jiangsu Engineering Research Center for Efficient Delivery System of TCM, School of Pharmacy, Nanjing University of Chinese Medicine, 138 Xianlin Avenue, Qixia District, Nanjing 210023, China; Jiangsu Engineering Research Center for Efficient Delivery System of TCM, School of Pharmacy, Nanjing University of Chinese Medicine, 138 Xianlin Avenue, Qixia District, Nanjing 210023, China; Department of Pathogen Biology, School of Medicine and Holistic Integrative Medicine, Nanjing University of Chinese Medicine, 138 Xianlin Avenue, Qixia District, Nanjing 210023, China; Jiangsu Engineering Research Center for Efficient Delivery System of TCM, School of Pharmacy, Nanjing University of Chinese Medicine, 138 Xianlin Avenue, Qixia District, Nanjing 210023, China

**Keywords:** Photosynthesis, Oxygen therapy, Hypoxia, Wound healing, Chronic wounds, Hypoxia environment

## Abstract

The hypoxic environment is among the most important factors that complicates the healing of chronic wounds, such as venous leg ulcers, pressure injuries and diabetic foot ulcers, which seriously affects the quality of life of patients. Various oxygen supply treatments are used in clinical practice to improve the hypoxic environment at the wound site. However, problems still occur, such as insufficient oxygen supply, short oxygen infusion time and potential biosafety risks. In recent years, artificial photosynthetic systems have become a research hotspot in the fields of materials and energy. Photosynthesis is expected to improve the oxygen level at wound sites and promote wound healing because the method provides a continuous oxygen supply and has good biosafety. In this paper, oxygen treatment methods for wounds are reviewed, and the oxygen supply principle and construction of artificial photosynthesis systems are described. Finally, research progress on the photosynthetic oxygen production system to promote wound healing is summarized.

Highlights

- The characteristics of chronic wounds and their relationship with hypoxic environments are reviewed and the current oxygen supply treatment methods used for wound treatment are summarized.
- The photosynthetic oxygen production system has good biological safety and can continuously supply oxygen to improve the hypoxic microenvironment. It provides an effective approach for the treatment of chronic wounds.
- The current research cases of a photosynthetic oxygen production system promoting chronic wound healing are summarized, and the advantages, limitations and prospects are discussed. It is hoped to provide ideas and methods for developing oxygen generation systems and promoting clinical transformation.

## Background

Chronic wounds refer to tissue injuries that fail to anatomically and functionally recovery through a series of orderly and continuous repair processes and have not healed or shown signs of healing for >30 days [1]. Common types of chronic wounds include venous leg ulcers, pressure injuries and diabetic foot ulcers [2]. Chronic wounds persist for a long duration, are difficult and costly to treat, seriously affect the patient’s quality of life, and increase the economic and care burden of patients.

**Figure 1:**
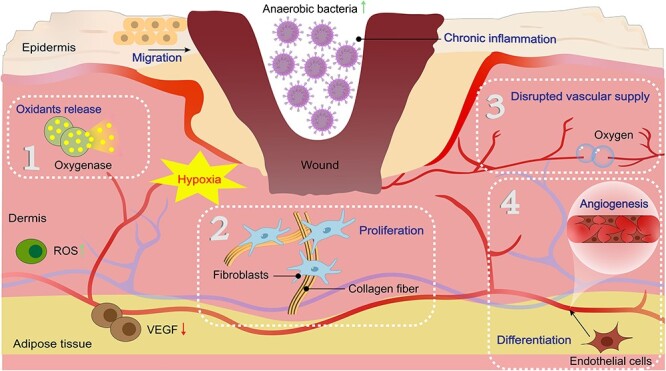
Relationship between chronic wounds and a hypoxic environment. (1) Oxygenase catalyzes the production of oxidants by consuming large amounts of oxygen. (2) Collagen formation and fibroblast proliferation are oxygen dependent. (3) A disrupted vascular supply immediately obstructs oxygen delivery to the wound. (4) Angiogenesis during endothelial cell differentiation also consumes large amounts of oxygen. *ROS* reactive oxygen species

Hypoxia is a clinical problem that urgently needs to be addressed in the treatment of chronic wounds. Chronic wounds exhibit hypoxia because the partial pressure of oxygen (pO_2_) at the center of the wound is usually lower than the critical threshold needed to fully support the enzymatic processes of tissue regeneration. When the pO_2_ at the wound site is <30 mmHg, healing is difficult [3,4]. Research has shown that oxygen is crucial for normal cell growth and respiration [5,6]. Although the mechanism by which oxygen functions at the cellular and molecular levels is unclear, the role of oxygen in wound healing is unquestionable. In the early stage of injury, the proliferation of anaerobic bacteria on the wound induces an inflammatory response [7,8], which triggers NADPH-linked oxygenase to catalyze the production of oxidants and consumes a large amount of oxygen [9–11]. With the continuous consumption of oxygen, local hypoxia develops at the wound site. This anoxic microenvironment intensifies the proliferation of anaerobic bacteria and persistent inflammation [12]. Moreover, the formation of collagen and the proliferation of fibroblasts consume oxygen. To repair damaged tissue, cellular metabolism is increased to further aggravate hypoxia [13]. After acute injury, the damaged vascular system immediately obstructs oxygen delivery to the wound, resulting in a decrease in oxygen tension at the wound site [10,11,14]. During angiogenesis, temporary hypoxia can initiate the wound healing process, but long-term hypoxia leads to decreased energy production and vascular endothelial growth factor (VEGF) levels, which hinder wound angiogenesis [8]. In addition, hypoxia promotes reactive oxygen species production, further exacerbating tissue damage and having adverse effects on wound healing [15]. In summary, each stage of wound progression and repair requires oxygen to accelerate cell proliferation and tissue remodeling. However, because the injured site cannot effectively transport and receive a certain concentration of oxygen, the imbalance between oxygen consumption and delivery leads to a malignant cycle of hypoxia at the wound site (Figure 1).

Therefore, providing an adequate oxygen supply is an effective strategy for improving the hypoxic wound environment and promoting wound repair. This review summarizes the current methods of oxygen supply therapy, focusing on the photosynthesis-based oxygen generation system in bionic oxygen therapy, including its composition, architecture and research progress in the treatment of chronic wounds. This review may help to inspire and promote the optimization and development of photosynthesis-based oxygen supply systems and provide a new method for clinical wound treatment.

## Review

### Oxygen supply therapy

Ideally, oxygen therapy should provide a sufficient and continuous supply of oxygen to the wound site, improve the pO_2_ of the wound, and achieve good biocompatibility and patient compliance. The existing oxygen supply therapy strategies mainly include direct oxygen delivery, chemical oxygen production and biomimetic oxygen supply systems. Direct oxygen delivery therapy uses equipment to directly deliver pure oxygen to the whole body or wound site [16,17]. Chemical oxygen generation strategies are artificial oxygen supply systems based on chemical reactions that can achieve oxygen delivery to the wound site through a suitable carrier [18–20]. The biomimetic oxygen supply system is based on photosynthesis in Nature, artificially simulating the morphology or function of leaves, chloroplasts or thylakoids and generating oxygen through photosynthesis [21]. Although the design principle and oxygen source are different, these strategies can improve the oxygen-depleted environment of the wound.

### Direct oxygen delivery therapy

#### Hyperbaric oxygen therapy

Hyperbaric oxygen therapy (HBOT) involves breathing 100% oxygen in a pressurized environment. This process must be performed in a specific chamber in which the air pressure is two to three times greater than normal [22]. HBOT has been used for wound treatment for >20 years [23], and is effective at increasing pO_2_ and dissolved oxygen in arterial blood [24]. Studies have shown that compared with standard wound treatment alone, HBOT-assisted treatment can promote wound metabolism and accelerate wound healing by regulating the collagen synthesis pathway [25], thus effectively reducing the risk of amputation in diabetic foot patients [26,27]. However, as a systemic oxygen supply strategy, HBOT may induce hyperoxia in tissues and produce toxic symptoms, such as ear injury, claustrophobia, sinus congestion and pressure [16,28]. In addition, HBOT is expensive, time-consuming and requires specific enclosed spaces, which limits its usefulness and compliance.

#### Topical oxygen therapy

Topical oxygen therapy (TOT) has improved the shortcomings of HBOT, as it directly supplies oxygen to the local hypoxic area through a continuous delivery or compression system, increasing the pO_2_ in the wound tissue and preventing adverse effects caused by systemic hyperoxia [29]. As an early form of TOT, topical pressurized oxygen therapy (TPOT) involves the use of a chamber or bag, which encloses the affected areas while administering 100% oxygen at a pressure that is slightly >1 atm. TPOT reduces costs, can be performed at home and minimizes the potential side effects associated with HBOT. However, the current reports on TPOT have mainly involved small, nonrandomized clinical studies [30] and larger clinical feedback and mechanistic studies are still needed. In addition, the toxicity of TPOT to the local wound endothelium has also been reported [8,31]. Therefore, a safe and standardized treatment protocol is urgently needed. Another method of treating TOT is topical continuous oxygen therapy (TCOT). This method does not require pressure or a chamber for administration. The portable unit produces a pure, continuous flow of oxygen 24 h a day for 7 days [32,33]. The absence of a pressure chamber increases the portability of TCOT. However, as with TPOT, larger studies are needed to thoroughly investigate and evaluate its clinical applicability [16,17]. Therefore, breaking through new oxygen sources and delivering oxygen conveniently and sustainably to wounds are the keys to the design of oxygen supply therapy systems. Notably, the form of oxygen also affects the delivery efficiency. Studies have shown that dissolved oxygen penetrates the skin more easily than gaseous oxygen, possibly because gaseous oxygen must overcome a liquid barrier before diffusing into cells [34].

#### Chemical oxygen production therapy

Many chemical reactions can produce oxygen. The oxygen-producing raw material is encapsulated in a suitable carrier to prepare the wound dressing [8]. Under certain conditions, oxidative reactions are triggered and oxygen is transported directly to the wound site [35]. This method of oxygen supply does not depend on equipment, which greatly improves the convenience of use. Chandra *et al.* [18] developed a wound dressing film that chemically generates oxygen *in situ* using sodium percarbonate and calcium peroxide to deliver oxygen to dermal wounds for 3 days. Wang *et al.* [19] developed a series of biocompatible and multifunctional hydrogels containing MnO_2_. MnO_2_ nanoparticles were added to convert endogenous hydrogen peroxide into oxygen. At present, the main oxidation-producing chemical reagents are metal oxides, and their gas production is often regulated by endogenous chemical reaction initiators. However, the residue of the reaction, especially some limited types of heavy metal ions, must be scrutinized and evaluated for safety [29,36,37].

#### Biomimetic oxygen supply systems

**Figure 2:**
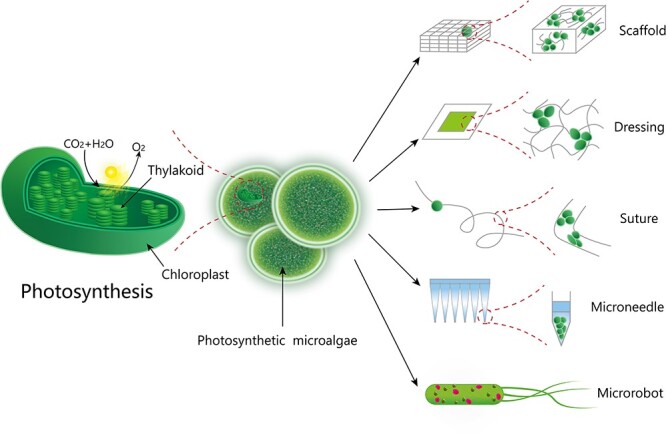
Principle of oxygen production through photosynthesis and composition of photosynthetic oxygen production systems

Bionics studies focus on artificially simulating the structure and function of living organisms and developing new devices, tools and techniques based on how they work. In Nature, the largest oxygen-producing system is photosynthesis. Photosynthesis is a process by which phototrophs convert light energy into chemical energy, accompanied by the release of oxygen. Complete photosynthesis consists of two stages: the light reaction and the dark reaction [38]. The former stage is light dependent and includes a series of events, such as light absorption, hydrolysis, oxygen release, and ATP and NADPH formation, which occur in the thylakoids of chloroplasts. The dark reaction, also known as nitrogen fixation, converts captured carbon dioxide into glucose. This stage is completed in the stroma of chloroplasts (Figure 2). Naturally transplanted or artificially constructed photosynthetic components are encapsulated into suitable wound dressings or devices through carriers, which form a bionic photosynthetic system [39]. This process is triggered by the endogenous signal of the lesion, completing all or part of the photosynthetic reaction, such as oxygen production. Therefore, the key to this strategy is to construct a photosynthetic oxygen generation system that mainly includes a photosynthetic reaction subject, carrier and light source [40,41]. The main body is an independent unit that can perform photosynthesis, and the carrier is a suitable place to maintain the stability and function of the main body. To ensure that the whole system works properly, an ideal light source is also necessary.

### Photosynthesis-based oxygen therapy

#### The main bodies of photosynthetic oxygen production systems

The main bodies that can produce oxygen through photosynthesis include photosynthetic microorganisms, chloroplasts or thylakoids, of which photosynthetic microorganisms are currently the most widely used. As an alternative to local oxygen therapy, photosynthetic microorganisms have been shown to perform well in various hypoxia-related diseases, such as tumors [42–45], stroke [46–49], ischemia injury [50,51] and wounds [52–54].

The most used photosynthetic microorganisms are microalgae and cyanobacteria [49]. Microalgae resources are abundant and easy to obtain, less raw material and space are required, and their photosynthetic efficiency is greater than that of land plants. Previous studies have demonstrated that microalgae have good biosafety *in vivo*, including low immunogenicity [55,56]. Therefore, various microalgae have been used in photosynthetic oxygen production experiments and have shown excellent performance [55,57]. At present, the most studied microalgae are Chlorella and *Chlamydomonas reinhardtii*. Chlorella species have a high artificial reproduction rate, an extremely fast growth rate, wide distribution and high photosynthetic efficiency. *Chlamydomonas reinhardtii* is widely distributed and nutritious. It has been recognized as “generally safe” by the US Food and Drug Administration and has been approved by the National Health Commission of China as a new food raw material. Studies have shown that *C. reinhardtii* microalgal cells can reduce tissue hypoxia [58], with good safety in animal models [56] and human patients [55]. The oxygen concentration released by microalgae under red light irradiation reaches 600 μM within 30 min [59]. Cell experiments have shown that the dissolved oxygen released by microalgae can promote the proliferation and epithelialization of human skin fibroblasts, which is conducive to chronic wound healing. The release of dissolved oxygen significantly enhances the migration and angiogenesis of human umbilical vein endothelial cells and does not induce an immune response [53]. Cyanobacteria, also known as blue–green algae, are different from bacteria because they contain thylakoids and photosynthetic pigments, which can produce oxygen through photosynthesis. Cyanobacteria are single-celled prokaryotes with simple structures and strong reproductive ability, so they are widely used in research on algal photosynthetic oxygen production [60]. Spirulina and *Synechococcus elongatus* are typical representatives [53,61].

#### Carriers of photosynthetic oxygen production systems

To facilitate clinical application, the photosynthetic main body needs to be encapsulated in a suitable carrier, which has the following main functions: (1) to maintain the stability of the photosynthetic main body; (2) to provide continuous oxygen production capacity; (3) to improve oxygen delivery efficiency and penetration depth; and (4) to exert synergistic healing effects. Representative carriers of the photosynthetic systems are shown in Figure 2.

Wound dressings are the most used medical supplies in wound care. The ideal dressing can protect a wound from secondary injury, form a suitable tissue repair microenvironment, and support cell growth and wound healing. There are various types of dressings, such as hydrogels, microspheres [62] and scaffolds [63]. Hydrogels are a 3D porous carrier with excellent absorption capacity and oxygen permeability. The structure of hydrogels is like that of the extracellular matrix, which is conducive to cell regeneration and wound healing [64]. Chen *et al.* [53] developed an oxygen-producing hydrogel patch made of live microalgae. The patch can continuously produce dissolved oxygen, and its penetration efficiency is >100 times that of gaseous oxygen. The hydrogel can also be formed into a scaffold through microfluid printing technology. Wound scaffolds not only provide sufficient space for cell growth and reproduction but also ensure efficient oxygen delivery [65].

In addition to conventional dressings, photosynthetic systems can be combined with other specific carriers. Generally, surgical sutures only provide mechanical sutures and have no biological function [52]. Thus, photosynthetic microalgae can be introduced into sutures, and functional sutures can not only seal the wound mechanically but also release oxygen continuously in the wound to accelerate wound healing. Microneedles, as a highly effective transdermal drug delivery method, can improve the permeability of the skin and are minimally invasive, painless and easy to use [66–68]. In addition, the microneedles can be loaded with a variety of active substances, including oxygen delivery carriers, to improve the anoxic environment of the wound [69]. Microrobots could carry active substances deep into wounds due to their autonomous mobility. Choi *et al.* [70] developed a microalgae-based biohybrid microrobot that can move autonomously at a speed of 33.3 μm·s^−1^ while generating oxygen to relieve hypoxia. In addition, the microrobot can effectively bind to inflammatory chemokines to regulate the immune response. Compared with other carriers, microrobots can better deliver therapeutic agents to deep within the wound.

In terms of carrier packaging and molding, various carriers are generated through individual preparation methods. In addition, the electrospinning, microfluidic and 3D printing technologies developed in recent years provide new ideas for the improved application of artificial photosynthetic systems in wound treatment. Microfluidic technology can convert hydrogels into hydrogel particles and deliver them as suspensions, granular hydrogels or a composite network within a bulk hydrogel. Encapsulation of cells into hydrogel particles can avoid the effects of shear force on cells caused by direct injection [71]. 3D printing has several advantages, including controllable geometry, customizable structure and adjustable function [72,73]. Porous scaffolds prepared by 3D printing technology can not only provide structural support for cell migration and the growth of tissues but also serve as effective carriers of therapeutic agents [74,75]. With 3D printing, *C. reinhardtii* can be embedded in a highly structured grid to achieve stable growth [76]. Compared to traditional methods, 3D printing offers far more flexibility in the structure and function of materials, and more process parameters can be set and optimized. For example, the number of fibers, the proportion of different fibers and their direction and weaving mode, and the concentration of therapeutic agents in each fiber can be adjusted to further affect the release of therapeutic agents [74]. Unicellular algae and cell cultures of higher plants can be integrated into 3D hydrogel scaffolds, achieving a new breakthrough in “green bioprinting” [77].

#### Light source

An effective light supply is a key condition for oxygen production during photosynthesis. The light parameters include wavelength, light intensity, irradiation position, irradiation time etc., which are mainly determined by the main photosynthetic body [78]. In addition, the effect of the interaction between the carrier and the main photosynthetic body on photosynthetic efficiency should be considered. Compared with other fields, the application scenarios of photosynthetic oxygen generation systems in the biomedical field may be more complex and diverse. For exposed skin or wounds, the light source choice is not limited; however, for some hidden or deep tissue areas, specific light supply devices need to be considered [79,80]. To date, the existing *in vivo* illumination strategies cannot generally meet the depth, area, wavelength and power requirements in different work scenarios, especially large-area illumination for deep targets [81]. Even with light from near-infrared I and II windows, it is difficult to reach deep organs and tissues using external sources [82]. A mainstream solution is directly delivering light sources to targets in the body [83]. Some researchers have used implantable near-field communication-based light-emitting diodes to wirelessly convert *in vitro* magnetic energy into optical energy [84] or upconversion nanoparticles to convert longwave light into shortwave light *in situ* [85]. Another study embedded flexible tapered optical fiber (TOF) into biodegradable transparent poly (4-methyl-ε-caprolactone) (PMCL) patches to form integrated optics. Diffraction at the TOF end and refraction at the air/TOF and air/PMCL interfaces together enhance the scattering of delivered optical energy, enabling a large area and deep penetration of luminescence without causing invasive damage to the target tissue [86].

#### Research progress in promoting wound healing via a photosynthetic oxygen production system

At present, progress in understanding the photosynthetic oxygen generation system in the field of wound treatment is increasing, which is mainly reflected by advances in the main body of photosynthesis and the application method, which will be discussed separately in this section (Table 1).

**Table 1:** Construction and function of photosynthetic oxygen supply system

Composition	Preparation formulation	Function	Ref.
*Chlorella*	Microneedle	Continuously produce O_2_	[69]
	Scaffold	Produce sustainable oxygen under light illumination	[54]
*Chlorella pyrenoidosa* and *Panax notoginsen*g saponins	Scaffold	Produce continuous oxygen, inhibit inflammation, promote angiogenesis and wound healing	[65]
*Chlorella pyrenoidosa* and *Bacillus subtilis*	Hydrogel	Continuously produce oxygen and destroy the colonized pathogenic bacteria	[87]
*Chlamydomonas reinhardtii*	Scaffold	Produce O_2_ through photosynthesis	[58]
	Hydrogel	Continuously produce oxygen under light and release human VEGF	[88]
	Sutures	Release oxygen and recombinant human growth factors (VEGF, PDGF-BB or SDF-1α)	[52]
*Synechococcus elongatus*	Hydrogel	Produce O_2_ through photosynthesis	[53]
	Scaffold	Produce oxygen and lymphangiogenic hyaluronic acid	[89]
*Spirulina platensis*	Hydrogel	Produce O_2_ and reactive oxygen species	[61]

*VEGF* vascular endothelial growth factor, *PDGF-BB *platelet-derived growth factor BB, *SDF-1α* stromal derived factor 1

#### Natural photosynthetic main body

To deliver oxygen to wounds, Chen *et al.* [53] filled the patch with 1 mm diameter hydrogel beads containing live microalgae, which can consume the pre-added carbonate and produce O_2_ through photosynthesis. The patch was filled with 1 mm diameter hydrogel beads containing live microalgae, which can consume the preadded carbonate and produce O_2_ through photosynthesis. The patch is lined with a hydrophilic polytetrafluoroethylene film with a pore size of 0.22 μm, which can allow bidirectional penetration of clean gas and water but prevent bacteria from passing through. The other side of the patch uses an impermeable polyurethane film as a back liner, forming an aerobic and moist sealing system between the dressing and the wound, which is conducive to tissue repair.

Schenck *et al.* [59] conducted a similar study in which green algae were injected into a collagen scaffold and directly implanted into full-thickness skin defects in mice. The results showed a greater degree of vascularization in the presence of microalgae. Hopfner *et al.* [58] implanted *C. reinhardtii* into a dermal collagen scaffold to improve the hypoxic response of fibroblasts through photosynthesis, providing oxygen independent of vascularization and perfusion, and good biocompatibility was attained. To verify the safety of the implantation of photosynthetic scaffolds in human patients, early phase 1 clinical trials were conducted [55]. Photosynthetic scaffolds for dermal regeneration were implanted in eight patients with full-thickness skin wounds. During 90 days of treatment, *C. reinhardtii* implanted in the scaffolds did not trigger harmful local or systemic immune responses, thus enabling complete tissue regeneration in humans and demonstrating the safety and feasibility of the scaffolds. The results of this study provide support for the clinical application of photosynthetic therapies.

Zhao *et al.* [69] proposed a separable microneedle (MN) loaded with active *Chlorella vulgaris* (Cv). CvMN consists of a polyvinyl acetate (PVA) substrate and gelatin methyl acryloyl group (GelMA) tips. Once CvMN is applied to diabetic wounds, the PVA basal layer is rapidly dissolved, while the GelMA tips remain in the skin. Through the photosynthesis of Cv, oxygen is continuously produced in an ecological way and released from CvMN in a controlled manner. The *in vivo* and *in vitro* experimental results showed that CvMNs can effectively promote cell proliferation, migration and angiogenesis and promote wound healing in diabetic mice with good biocompatibility.

Wang *et al.* [54] employed a microfluidic-assisted bioprinting strategy to directly deposit living microalgae-laden hollow fibrous (MA-HF) scaffolds into defect sites to promote wound closure. Owing to the photosynthesis performed by embedded living microalgae (*Chlorella pyrenoidosa*), the scaffolds could produce sustainable oxygen under light illumination, which facilitated cell proliferation, migration and differentiation even under hypoxic conditions. The results showed that MA-HF scaffolds directly printed into chronic diabetic wounds can significantly accelerate wound healing by relieving local hypoxia and promoting angiogenesis and extracellular matrix (ECM) synthesis after irradiation. The advantage of this scaffold is that it can adapt to irregularly shaped wounds and promote their healing, providing a promising strategy for the application of tissue engineering in wound repair and treatment.

#### Engineered photosynthetic main body

Chíavez *et al.* [89] developed a viable photosynthetic scaffold by seeding transgenic *Synechococcus* sp. PCC 7002 cyanobacteria (SynHA) into collagen-based dermal regeneration materials that can produce oxygen and lymphangiogenic hyaluronic acid. SynHA supported the viability of cocultured lymphatic endothelial cells under hypoxic conditions by providing them with photosynthetic-derived oxygen, while cyanobacteria-derived hyaluronic acid increased the lymphangiogenic capacity of lymphatic endothelial cells. Since tissue hypoxia and impaired lymphatic drainage are two key factors that directly affect wound healing, this photosynthetic scaffold could become a treatment option for chronic wounds.

In addition to producing oxygen through photosynthesis, engineered photosynthesis systems can release other biologically active molecules. Corrales-Orovio *et al.* [88] have prepared genetically engineered microalgae that can continuously produce oxygen under light and release human VEGF, thus promoting angiogenesis. Centeno-Cerdas *et al.* [52] developed photosynthesis-based sutures with biological activity. When transgenic photosynthetic microalgae were inoculated into the suture, oxygen and recombinant human growth factors (VEGF, PDGF-BB or SDF-1α) could be released stably at the suture site, and their mechanical stress and freezing resistance could meet clinical needs.

#### In combination with other molecular therapeutic agents

The photosynthetic system is often combined with other molecular therapeutic agents to provide oxygen while superposing antibacterial, anti-inflammatory, oxidation and other functions to achieve synergistic therapy. Li *et al.* [61] used carboxymethyl chitosan to coat Spirulina platensis (SP) to generate a SP gel. During treatment, SP maintained a sustained oxygen-producing capacity and increased the oxygen level at the wound site. At the same time, the natural photosensitizer chlorophyll can produce reactive oxygen species, which can kill bacteria at infected sites. Algae containing large amounts of photosensitizers can synergically promote wound healing by improving hypoxia and resisting infection. Wang *et al.* [65] recently constructed a Chinese herbal medicine scaffold to provide a sustainable oxygen and nutrient supply for promoting wound healing. *Chlorella pyrenoidosa* and *Panax notoginsen*g saponin, a Chinese herbal medicine that inhibits inflammation, promotes angiogenesis and promotes wound healing in diabetes, were encapsulated in the scaffold. Encapsulated *Panax notoginsen*g saponin can be slowly released from the scaffold to promote cell adhesion, proliferation, migration and angiogenesis. Living algae use photosynthesis to produce continuous amounts of oxygen in the light, alleviating cell hypoxia. *In vivo* experiments have shown that functional scaffolds can effectively relieve local hypoxia in diabetic mice, promote angiogenesis and thus accelerate wound healing.

#### In combination with aerobic bacteria

Inspired by bacterial colonization resistance, Chen *et al.* [87] encapsulated live *C. pyrenoidosa* and the probiotic *Bacillus subtilis* in a temperature-sensitive hydrogel to continuously supply oxygen to wounds and exert anti-infection effects. The hydrogels were liquid at low temperatures but solidified rapidly at body temperature to achieve retention at the wound site. The results showed that *C. pyrenoidosa* could continuously produce oxygen to relieve wound hypoxia and simultaneously support the proliferation of *B. subtilis*. *Bacillus subtilis* can destroy colonized pathogenic bacteria and collaboratively promote the healing of diabetic infected wounds.

## Conclusions

Chronic wounds are difficult to heal due to severe hypoxia and persistent inflammation, which seriously affects the health and life of patients. The hypoxic environment of wounds can be improved by existing oxygen supply treatments, but achieving a portable, durable oxygen source remains a challenge. Photosynthesis is the largest oxygen supply pathway in Nature, is regulated by light radiation and is a promising oxygen-producing strategy. Although positive feedback has been obtained in laboratory and small clinical sample experiments, the current photosynthetic oxygen supply system must be improved, mainly as follows. (1) Light penetration is insufficient, especially for complex wound sites that light cannot easily reach, so it is necessary to build a light source guidance device. (2) The main body of photosynthesis with sufficient oxygen supply capacity and portability is the core component. (3) Implant devices must exhibit adequate biocompatibility, especially when applied to trauma sites. (4) Controlled adjustment of oxygen supply flow must be achieved to prevent oxygen overload. In addition, some industrialization issues, such as the storage stability, photosynthetic efficiency of materials and large-scale production of preparations, should be considered. After confirming that the photosynthetic oxygen generation system can indeed provide controlled oxygen to the hypoxic site and reach therapeutic concentrations, researchers should explore the difficulties and solutions of its clinical transformation.
